# Timeliness of diagnostic evaluation for postmenopausal bleeding: A retrospective cohort study using claims data

**DOI:** 10.1371/journal.pone.0289692

**Published:** 2023-09-08

**Authors:** Xiao Xu, Ling Chen, Marcella Nunez-Smith, Mitchell Clark, Jason D. Wright

**Affiliations:** 1 Department of Obstetrics, Gynecology and Reproductive Sciences, Yale School of Medicine, New Haven, Connecticut, United States of America; 2 Yale Cancer Outcomes, Public Policy and Effectiveness Research (COPPER) Center, Yale School of Medicine, New Haven, Connecticut, United States of America; 3 Department of Obstetrics and Gynecology, Columbia University Vagelos College of Physicians and Surgeons, New York, New York, United States of America; 4 Department of Internal Medicine, Yale School of Medicine, New Haven, Connecticut, United States of America; Public Library of Science, UNITED KINGDOM

## Abstract

**Background:**

Postmenopausal bleeding (PMB) is a common gynecologic condition. Although it can be a sign of uterine cancer, most patients have benign etiology. However, research on quality of diagnostic evaluation for PMB has been limited to cancer patients. To extend this research, we examined the timeliness of diagnostic evaluation for PMB among patients with benign conditions.

**Methods:**

Using the 2008–2019 MarketScan Research Databases, we identified 499176 patients (456741 with commercial insurance and 42435 with Medicaid insurance) who presented with PMB but did not have gynecologic cancer. For each patient, we measured the time from their PMB reporting to the date of their first diagnostic procedure. The association between patient characteristics and time to first diagnostic procedure was examined using Cox proportional hazards models (for the overall sample and then stratified by insurance type).

**Results:**

Overall, 54.3% of patients received a diagnostic procedure on the same day when they reported PMB and 86.6% received a diagnostic procedure within 12 months after reporting PMB. These percentages were 39.4% and 77.1%, respectively, for Medicaid patients, compared to 55.7% and 87.4%, respectively, for commercially insured patients (p<0.001 for both). Medicaid patients had an 18% lower rate of receiving a diagnostic procedure at any given time point than commercially insured patients (adjusted hazard ratio = 0.82, 95% CI: 0.81–0.83). Meanwhile, older age and non-gynecologic comorbidities were associated with a lower rate whereas concomitant gynecologic conditions and recent use of preventive care were associated with a higher rate of receiving diagnostic procedures. Analysis stratified by insurance type identified additional risk factors for delayed diagnostic procedures (e.g., non-metropolitan versus metropolitan location for commercially insured patients and Black versus White race for Medicaid patients).

**Conclusion:**

A sizable proportion of patients did not receive prompt diagnostic evaluation for PMB. Both clinical and non-clinical factors could affect timeliness of evaluation.

## Introduction

Postmenopausal bleeding (PMB) is a common condition, accounting for 4.3% of problem-based visits to gynecologists [1]. As PMB can be a warning sign of uterine cancer [2], careful diagnostic evaluation is crucial for identifying the underlying causes of bleeding, and the American College of Obstetricians and Gynecologists (ACOG) calls for a “prompt and efficient evaluation” [3]. However, few studies have examined the timeliness of diagnostic evaluation for patients presenting with PMB, leaving the quality of care in this area largely unknown.

Moreover, the limited data available so far were limited to PMB among patients with uterine cancer [4, 5]. Although about 9% of patients with PMB in general and 5% of those in North America have uterine cancer [6], PMB is most commonly caused by benign conditions (e.g., uterine fibroids, polyps) [7, 8]. It is unclear how postmenopausal patients with benign conditions are evaluated when presenting with vaginal bleeding, who account for the vast majority of patients with PMB. For these patients, despite having benign conditions, timely evaluation is still important for identifying the etiology of bleeding and determining appropriate treatment/management plans.

Even less is known about factors that influence the timeliness of diagnostic evaluation for PMB. In particular, research suggests that patients with Medicaid insurance, as opposed to commercial insurance, tend to experience greater delay in care access [9–11]. Medicaid is a joint federal and state insurance program in the United States for people with limited income. Even though many states expanded its eligibility criteria in recent years, adult Medicaid beneficiaries without dependent children usually have income at or below 138% of the federal poverty level [12]. Due to issues such as lower payment rate and lower physician participation rate, Medicaid patients reportedly experience greater barrier to care [13]. Therefore, insurance type may affect timeliness of evaluation for PMB and warrants close examination.

To address these knowledge gaps, we analyzed large healthcare databases in the United States to examine the timeliness of diagnostic evaluation in a large sample of patients who presented with PMB but did not have gynecologic cancer. We also assessed the distribution of different types of diagnostic procedures used and examined how insurance type and other factors influenced the timeliness of diagnostic evaluation. The findings can inform opportunities for targeted interventions to improve quality of care.

## Materials and methods

### Data source and sample

We used the 2008–2019 IBM^®^ MarketScan^®^ Research Databases, which include a family of datasets integrating longitudinal claims data for a large sample of individuals with commercial insurance and Medicaid insurance in the United States [14]. Individuals with commercial insurance in the data included employees and retirees of all ages, as well as their spouse and dependents, with employer-sponsored private health insurance (persons age 65 or older were limited to those with both commercial insurance and Medicare coverage, which is a federal health insurance program for the elderly). Individuals with Medicaid insurance in the data came from multiple de-identified states and included Medicaid enrollees of all ages. Although availability of demographic variables differed for patients with different types of insurance, encounter-level diagnosis and procedure information for a complete record of all medical services was available for all individuals included, regardless of insurance type.

We identified female patients who had at least one insurance claim with a diagnosis code of PMB. Their first PMB claim was referred to as the index PMB. Patients were included in this study if they met the following criteria: 1) were 50 years of age or older at the time of index PMB, 2) had continuous insurance coverage from 9 months before through 12 months after the index PMB (to facilitate measurement of medical history and diagnostic process), 3) did not have a hysterectomy prior to the index PMB (to ensure a need for evaluation of the uterus), and 4) did not have a diagnosis of gynecologic cancer prior to the index PMB or within the subsequent 12 months (to focus on evaluation of PMB among non-cancer patients). To avoid an overly restrictive continuous enrollment period that might limit representativeness of the sample (especially for Medicaid enrollees who are particularly at risk for churning in insurance coverage [15]), we used a 9-month pre-PMB window to measure patients’ medical history. International Classification of Diseases (ICD) diagnosis/procedure codes and Current Procedural Terminology (CPT) codes were used to identify PMB, hysterectomy, and gynecologic cancers (S1 Table). This study was reviewed by the Columbia University Human Research Protection Office Institutional Review Board and was determined not human subjects research as it only involved secondary analysis of de-identified data (protocol number: AAAS2158; date of determination: December 19, 2018).

### Outcome measure

Our outcome measure was the time from the date of a patient’s index PMB to the date when they received their first diagnostic procedure. This included any of the following diagnostic procedures: endometrial biopsy, dilation and curettage, hysteroscopy, transvaginal/pelvic ultrasound, and pelvic magnetic resonance imaging (MRI). These procedures have been recommended by professional organizations as appropriate for evaluation of abnormal vaginal bleeding in various clinical contexts [2, 3, 16]. We measured each patient’s receipt of these diagnostic procedures from the date of their index PMB until 12 months afterwards using ICD and CPT procedure codes (S1 Table). Patients who did not receive any of these diagnostic procedures at the end of the observation period (i.e., 12 months after the index PMB) were considered censored.

### Covariates

For each patient, we categorized their type of health insurance as commercial insurance or Medicaid insurance. For both commercially insured patients and Medicaid patients, we were able to measure their age, presence of gynecologic conditions (leiomyoma, benign neoplasms or cysts, endometrial hyperplasia, cervical abnormality), non-gynecologic comorbidities (obesity, non-gynecologic cancer, number of other Elixhauser comorbidities [17]), use of preventive care, and the year of their index PMB. Presence of gynecologic conditions and comorbidities were measured based on ICD diagnosis codes on claims in the 9 months prior to and through the date of index PMB (S1 Table). Use of preventive care measured whether a patient received any of the following in the 9 months prior to the date of index PMB: wellness visits, preventive physical/pelvic exams, mammography screening, cervical cancer screening, and colorectal cancer screening. Use of these preventive services was identified based on CPT/Healthcare Common Procedure Coding System (HCPCS) codes on insurance claims (S1 Table). It served as a surrogate indicator for a patient’s access to and preference for preventive care, even though the 9 months window did not fully capture all use of preventive services.

For commercially insured patients, our data also had information on their geographic region (northeast, north central, south, west, or unknown) and metropolitan statistical area (MSA) location (yes, no, or unknown). For Medicaid patients, although information on geographic region and MSA location was not available, we were able to measure patients’ race and whether their Medicaid insurance plan used capitated versus fee-for-serve payment model. Race (Black, Hispanic, White, other, or unknown) was derived from the Medicaid enrollment profile and presumably reflect patients’ self-report.

### Statistical analysis

We summarized patient characteristics using descriptive statistics for the overall sample and stratified by type of health insurance. Patients with missing data on a given characteristic were categorized as “unknown” and were retained in analysis. Characteristics of patients with commercial insurance were compared with those with Medicaid insurance using chi-square tests for categorical variables and Wilcoxon rank-sum tests for continuous variables.

Time to first diagnostic procedure was summarized using cumulative incidence curves and compared between patients who had commercial insurance versus Medicaid insurance using log-rank test. We also compared the proportion of patients who received a diagnostic procedure on the same day of their index PMB, as well as within 12 months after the index PMB, between patients who had commercial insurance and those who had Medicaid insurance using chi-square tests. Comparisons were made for the overall proportion (i.e., any of the diagnostic procedures) and the proportion by different types of diagnostic procedure (e.g., endometrial biopsy, transvaginal/pelvic ultrasound, or hysteroscopy).

We estimated a multivariable Cox proportional hazards regression model to examine the association between various patient characteristics and time to first diagnostic procedure. The model adjusted for type of insurance (Medicaid versus commercial insurance), patient age, presence of gynecologic conditions, non-gynecologic comorbidities, use of preventive care, and the year of index PMB. To draw on additional measures of patient characteristics that were only available for commercially insured patients (geographic region and MSA location) or Medicaid patients (race and payment model) respectively, we also estimated separate Cox proportional hazards regressions stratified by insurance type and included these additional characteristics as covariates. Proportional hazards assumption in these models were assessed and confirmed by visual inspection of the log-minus-log plot and Shoenfeld residuals.

All statistical tests were two-sided. We used p values less than 0.05 as the cutoff for determining statistical significance. Analysis was conducted using SAS Studio 3.71 (SAS Institute Inc., Cary, NC). We followed the Strengthening the Reporting of Observational Studies in Epidemiology (STROBE) reporting guideline.

## Results

Our sample included a total of 499176 patients who newly presented with PMB and did not have gynecologic cancer or prior hysterectomy (n = 456741 had commercial insurance and n = 42435 had Medicaid insurance) (S1 Fig). Compared to patients with commercial insurance in the sample, those with Medicaid insurance tended to be older (e.g., 28.4% versus 45.2% were ≥60 years of age, p<0.001) and were more likely to be obese (9.1% versus 27.7%, p<0.001) (Table 1). Medicaid patients also had more comorbidities than commercially insured patients (median: 3 versus 1, p<0.001) and were less likely to have received preventive care in the previous 9 months (23.6% versus 60.2%, p<0.001). Although a lower proportion of Medicaid than commercially insured patients had benign gynecologic conditions, such as leiomyoma (11.7% versus 12.7%), benign neoplasms or cysts (7.7% versus 9.9%), and endometrial hyperplasia (2.5% versus 2.9%) (p<0.001 for all), the magnitude of these differences was small.

**Table 1:** Sample characteristics.

Characteristic	Overall Sample (N = 499176)		Stratified by Type of Health Insurance				
			Commercial Insurance (N = 456741)		Medicaid Insurance (N = 42435)		*P* value
	N	%	N	%	N	%	
Age, in years							<0.001
50–59	350478	70.2	327216	71.6	23262	54.8	
60–69	105540	21.1	94024	20.6	11516	27.1	
70–79	29035	5.8	24214	5.3	4821	11.4	
≥80	14123	2.8	11287	2.5	2836	6.7	
Race and ethnicity							NA
Black	-^a^	-^a^	-^b^	-^b^	16285	38.4	
Hispanic	-^a^	-^a^	-^b^	-^b^	834	2.0	
Unknown	-^a^	-^a^	-^b^	-^b^	4247	10.0	
White	-^a^	-^a^	-^b^	-^b^	20295	47.8	
None of the above	-^a^	-^a^	-^b^	-^b^	774	1.8	
Region							NA
Northeast	-^a^	-^a^	108747	23.8	-^c^	-^c^	
North central	-^a^	-^a^	98940	21.7	-^c^	-^c^	
South	-^a^	-^a^	162039	35.5	-^c^	-^c^	
West	-^a^	-^a^	79801	17.5	-^c^	-^c^	
Unknown	-^a^	-^a^	7214	1.6	-^c^	-^c^	
Metropolitan statistical area							NA
No	-^a^	-^a^	56115	12.3	-^c^	-^c^	
Yes	-^a^	-^a^	384966	84.3	-^c^	-^c^	
Unknown	-^a^	-^a^	15660	3.4	-^c^	-^c^	
Type of Medicaid plan							NA
Fee-for-service	-^a^	-^a^	-^b^	-^b^	28874	68.0	
Capitated	-^a^	-^a^	-^b^	-^b^	13561	32.0	
Leiomyoma	62837	12.6	57878	12.7	4959	11.7	<0.001
Benign neoplasms or cysts	48423	9.7	45150	9.9	3273	7.7	<0.001
Endometrial hyperplasia	14155	2.8	13076	2.9	1079	2.5	<0.001
Cervical abnormality	25383	5.1	23269	5.1	2114	5.0	0.31
Obesity	53439	10.7	41674	9.1	11765	27.7	<0.001
Non-gynecologic cancer	42434	8.5	38927	8.5	3507	8.3	0.07
Number of other Elixhauser comorbidities, median (IQR)	1	(0–2)	1	(0–2)	3	(2–5)	<0.001
Use of preventive care	284981	57.1	274974	60.2	10007	23.6	<0.001

IQR = interquartile range; NA = not applicable.

^a^ This variable was only available for patients with commercial insurance or only available for patients with Medicaid insurance.

^b^ This variable was not available for patients with commercial insurance.

^c^ This variable was not available for patients with Medicaid insurance.

**Fig 1:**
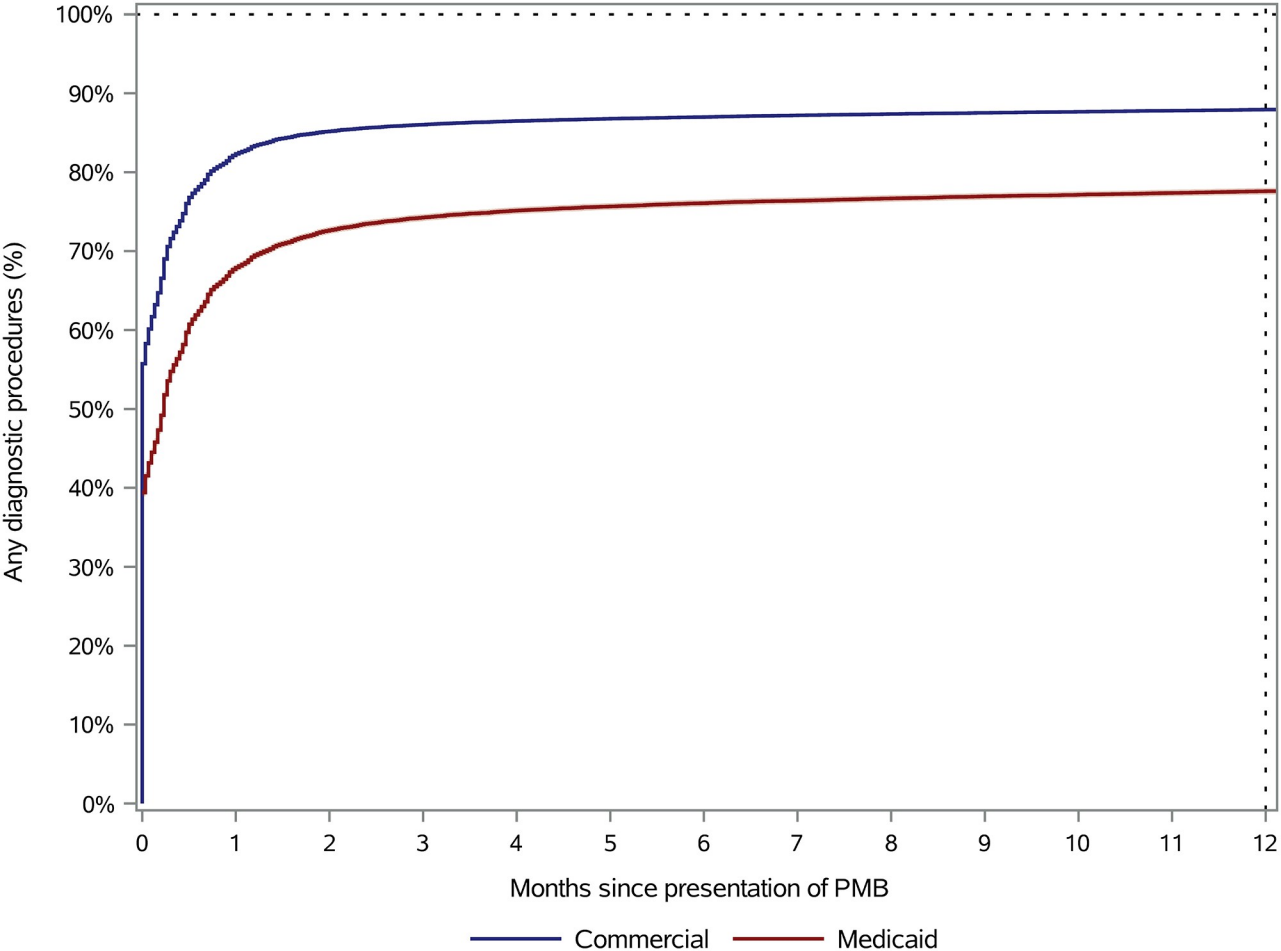
Time to first diagnostic procedure after presentation of postmenopausal bleeding, by type of insurance. PMB = postmenopausal bleeding. Log-rank test: p<0.001.

Overall, 54.3% (271251/499176) of patients received a diagnostic procedure on the same day when they reported PMB and 86.6% (432073/499176) received a diagnostic procedure within 12 months after PMB reporting. Fig 1 shows the cumulative incidence curve for receipt of diagnostic procedures over time after a patient reported PMB (stratified by type of insurance). On the day of index PMB, 39.4% (16724/42435) of Medicaid patients, as opposed to 55.7% (254527/456741) of commercially insured patients, received a diagnostic procedure (p<0.001). At each subsequent time point, a lower proportion of Medicaid patients received a diagnostic procedure than commercially insured patients (log-rank test p<0.001). Among patients who did receive a diagnostic procedure within 12 months after reporting PMB, the median (interquartile range) time to first diagnostic procedure was 0 (0, 13) days for Medicaid patients and 0 (0, 6) days for commercially insured patients (p<0.001).

**Fig 2:**
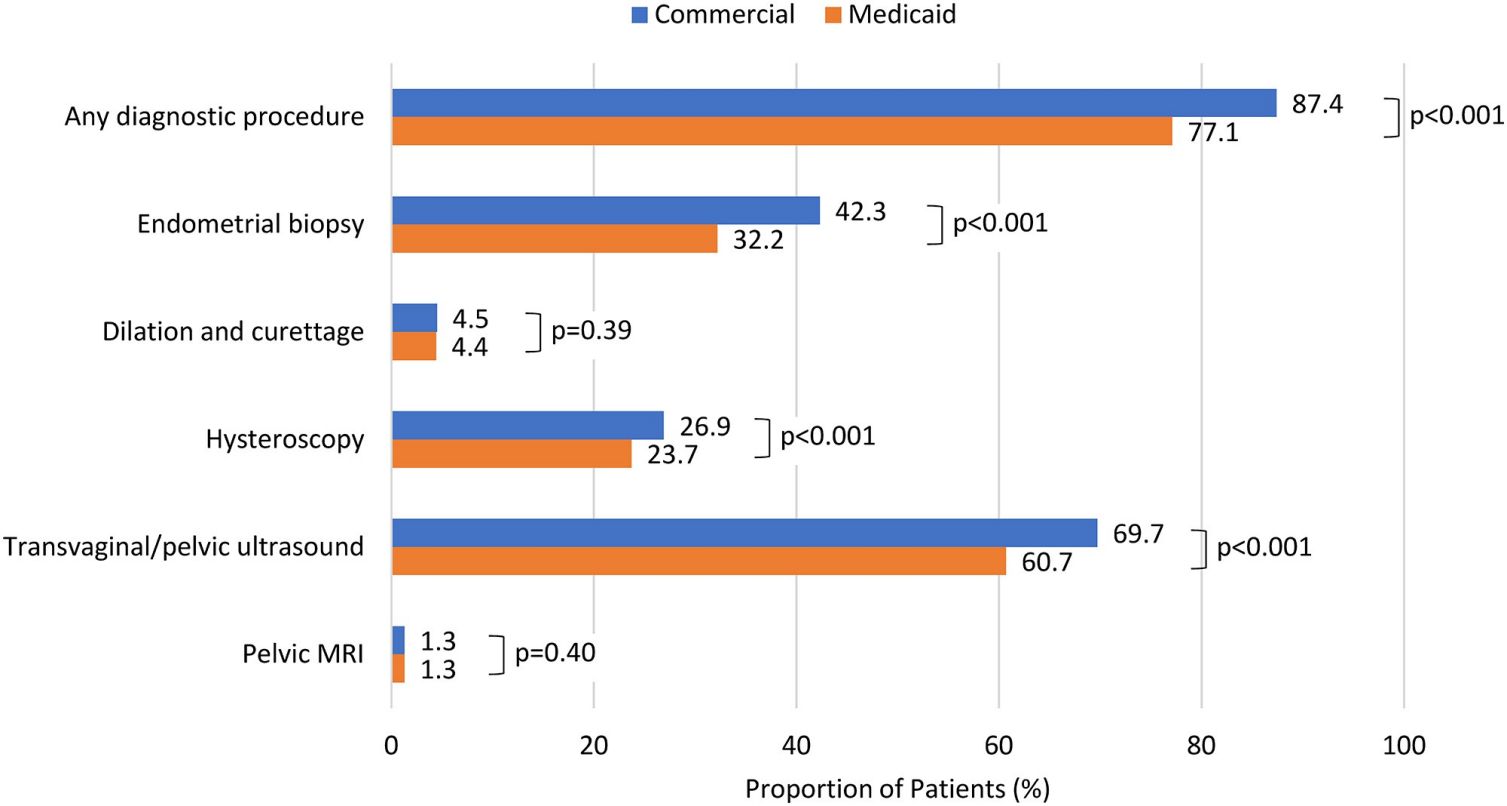
Type of diagnostic procedure received within 12 months after presentation of postmenopausal bleeding, by type of insurance. MRI = magnetic resonance imaging.

By 12 months after the index PMB, 77.1% (32735/42435) of patients with Medicaid insurance, in comparison to 87.4% (399338/456741) of patients with commercial insurance, received a diagnostic procedure (p<0.001) (Fig 2). During the 12 months after index PMB, transvaginal/pelvic ultrasound was the most frequently used diagnostic procedure, received by 60.7% of patients with Medicaid insurance and 69.7% of patients with commercial insurance (p<0.001). This was followed by endometrial biopsy (received by 32.2% of Medicaid patients and 42.3% of commercially insured patients, p<0.001) and hysteroscopy (received by 23.7% of Medicaid patients and 26.9% of commercially insured patients, p<0.001). Dilation and curettage and pelvic MRI were less frequently used, and their utilization rates did not differ significantly between patients with Medicaid and commercial insurance.

In multivariable regression analysis for the overall sample, the association between type of insurance and time to first diagnostic evaluation remained statistically significant even after adjusting for patient age, gynecologic and non-gynecologic comorbidities, use of preventive care, and the year of index PMB (Table 2). Compared to commercially insured patients, Medicaid patients had an 18% lower rate of receiving a diagnostic procedure at any given time point (adjusted hazard ratio [HR]: 0.82, 95% confidence interval [CI]: 0.81–0.83).

**Table 2:** Multivariable regression analysis of the association between patient characteristics and time to first diagnostic procedure.

Characteristic	Overall Sample	Stratified by Type of Health Insurance	
		Commercial Insurance	Medicaid Insurance
	Adjusted HR (95%CI)	Adjusted HR (95%CI)	Adjusted HR (95%CI)
Type of insurance			
Commercial	Referent	NA	NA
Medicaid	**0.82 (0.81–0.83)**	NA	NA
Age, in years			
50–59	Referent	Referent	Referent
60–69	**0.95 (0.94–0.96)**	**0.947 (0.940–0.955)**	0.98 (0.95–1.002)
70–79	**0.85 (0.84–0.86)**	**0.83 (0.82–0.84)**	**0.92 (0.89–0.96)**
≥80	**0.72 (0.70–0.73)**	**0.70 (0.68–0.71)**	**0.79 (0.76–0.83)**
Race and ethnicity			
Black	-^a^	-^b^	**0.92 (0.90–0.94)**
Hispanic	-^a^	-^b^	1.07 (0.99–1.16)
Unknown	-^a^	-^b^	1.00 (0.96–1.04)
White	-^a^	-^b^	Referent
None of the above	-^a^	-^b^	1.01 (0.93–1.09)
Region			
Northeast	-^a^	Referent	
North central	-^a^	1.00 (0.99–1.01)	-^c^
South	-^a^	**0.95 (0.94–0.96)**	-^c^
West	-^a^	**0.885 (0.876–0.894)**	-^c^
Unknown	-^a^	**0.90 (0.87–0.93)**	-^c^
Metropolitan statistical area			
No	-^a^	Referent	
Yes	-^a^	**1.01 (1.004–1.02)**	-^c^
Unknown	-^a^	**1.07 (1.04–1.10)**	-^c^
Type of Medicaid plan			
Fee-for-service	-^a^	-^b^	Referent
Capitated	-^a^	-^b^	**1.04 (1.01–1.06)**
Leiomyoma			
No	Referent	Referent	Referent
Yes	**1.15 (1.14–1.16)**	**1.14 (1.13–1.15)**	**1.28 (1.24–1.32)**
Benign neoplasms or cysts			
No	Referent	Referent	Referent
Yes	**1.21 (1.19–1.22)**	**1.19 (1.18–1.21)**	**1.40 (1.35–1.46)**
Endometrial hyperplasia			
No	Referent	Referent	Referent
Yes	**1.19 (1.17–1.21)**	**1.18 (1.16–1.20)**	**1.22 (1.14–1.30)**
Cervical abnormality			
No	Referent	Referent	Referent
Yes	0.990 (0.977–1.003)	0.99 (0.97–1.001)	1.02 (0.97–1.07)
Obesity			
No	Referent	Referent	Referent
Yes	1.00 (0.99–1.01)	0.99 (0.98–1.002)	**1.06 (1.04–1.09)**
Non-gynecologic cancer			
No	Referent	Referent	Referent
Yes	**1.03 (1.02–1.04)**	**1.03 (1.02–1.04)**	1.01 (0.97–1.05)
Number of other Elixhauser comorbidities			
Increase in one comorbidity	**0.982 (0.980–0.984)**	**0.980 (0.977–0.982)**	**0.989 (0.984–0.994)**
Preventive care			
No	Referent	Referent	Referent
Yes	**1.187 (1.179–1.194)**	**1.18 (1.17–1.19)**	**1.15 (1.12–1.18)**
Year when postmenopausal bleeding was reported			
2008	Referent	Referent	Referent
2009	1.01 (0.99–1.03)	1.02 (0.997–1.03)	0.96 (0.88–1.06)
2010	1.02 (0.998–1.03)	1.02 (0.999–1.04)	0.97 (0.89–1.07)
2011	**1.02 (1.0002–1.04)**	**1.02 (1.002–1.04)**	0.97 (0.88–1.07)
2012	1.02 (0.9998–1.04)	**1.02 (1.005–1.04)**	0.98 (0.89–1.08)
2013	**1.02 (1.005–1.04)**	**1.03 (1.01–1.05)**	0.97 (0.89–1.06)
2014	1.00 (0.99–1.02)	1.01 (0.99–1.03)	0.96 (0.88–1.06)
2015	0.99 (0.98–1.01)	0.99 (0.98–1.01)	0.96 (0.88–1.05)
2016	0.99 (0.97–1.01)	0.99 (0.97–1.01)	0.98 (0.90–1.08)
2017	1.00 (0.98–1.02)	0.98 (0.96–1.01)	1.01 (0.93–1.11)
2018	0.98 (0.96–1.003)	**0.97 (0.95–0.99)**	0.99 (0.91–1.09)

CI = confidence interval; HR = hazard ratio; NA = not applicable.

Bold font inside the table indicates statistically significant results.

^a^ This variable was only available for patients with commercial insurance or only available for patients with Medicaid insurance and hence was excluded from the regression model for the overall sample.

^b^ This variable was not available for patients with commercial insurance and hence was excluded from the regression model for commercially insured patients.

^c^ This variable was not available for patients with Medicaid insurance and hence was excluded from the regression model for Medicaid patients.

In addition, patients who were older in age (e.g., adjusted HR for age 70–79 versus age 50–59: 0.85, 95% CI: 0.84–0.86) or had more benign non-gynecologic comorbidities (adjusted HR associated with each additional comorbidity: 0.982, 95% CI: 0.980–0.984) had a lower rate of receiving diagnostic procedures at any given time point (Table 2). In contrast, those who had other gynecologic conditions (e.g., adjusted HR for leiomyoma: 1.15, 95% CI: 1.14–1.16) or used preventive care in the previous 9 months (adjusted HR: 1.187, 95% CI: 1.179–1.194) had a higher rate of receiving diagnostic procedures at any given time point. These associations remained similar when patients with commercial and Medicaid insurance were analyzed separately.

However, some other factors were found to influence the timeliness of diagnostic evaluation differently for commercially insured and Medicaid insured patients (Table 2). For example, in regression analysis stratified by type of insurance, obesity was only associated with a higher rate of receiving diagnostic procedures at any given time point among patients with Medicaid insurance (adjusted HR: 1.06, 95% CI: 1.04–1.09), but not among patients with commercial insurance (adjusted HR: 0.99, 95% CI: 0.98–1.002). In contrast, having non-gynecologic cancer was associated with a higher rate of receiving diagnostic procedures at any given time point among patients with commercial insurance (adjusted HR: 1.03, 95% CI: 1.02–1.04) but not among patients with Medicaid insurance (adjusted HR: 1.01, 95% CI: 0.97–1.05).

Regression analysis stratified by type of insurance also informed the role of some patient characteristics that were only available in data for the commercially insured or Medicaid insured subsample. Among commercially insured patients, those located in the south (adjusted HR: 0.95, 95% CI: 0.94–0.96) or the west (adjusted HR: 0.885, 95% CI: 0.876–0.894) had a lower rate of receiving diagnostic procedures at any given time point than those located in the northeast, whereas location in an MSA was associated with a higher rate of receiving diagnostic procedures (adjusted HR: 1.01, 95% CI: 1.004–1.02). Among Medicaid patients, Black race was associated with a lower rate of receiving diagnostic procedures at any given time point, compared to White race (adjusted HR: 0.92, 95% CI: 0.90–0.94), whereas enrollment in a capitated Medicaid plan was associated with a higher rate of receiving diagnostic procedures than enrollment in a fee-for-service Medicaid plan (adjusted HR: 1.04, 95% CI: 1.01–1.06).

## Discussion

Distinct from prior research focusing on cancer patients, this study filled an important gap in literature by informing the quality of diagnostic evaluation for PMB in the large number of patients without cancer. We delineated patterns of diagnostic evaluation for non-cancer patients with PMB and found that a sizable proportion of patients did not receive prompt diagnostic evaluation. In addition to clinical factors (e.g., patient age and comorbidities), non-clinical factors such as type of insurance, geographic region/location, and race were also associated with a patient’s risk of experiencing delayed evaluation. These findings pointed to several areas needing more clinical, policy, and research attention.

In clinical practice for PMB, uterine cancer should be assumed “until proven otherwise” [18, 19]. The ACOG recommends “prompt” evaluation to appropriately diagnose or rule out cancer [3]. Yet we found that only 39.4–55.7% of patients received a diagnostic procedure on the same day of PMB presentation and 77.1–87.4% received a diagnostic procedure within 12 months after PMB presentation. This highlights a need to improve the timeliness of PMB evaluation. Even for patients without cancer, delay in diagnostic evaluation of PMB could hinder appropriate management of their underlying condition. PMB may be caused by a variety of etiologies such as polyp, leiomyoma, endometritis, cervicitis, or atrophy of the vagina, which necessitates different treatment regimens (e.g., antibiotics, hysteroscopic procedures, endometrial ablation, and hysterectomy) [8]. Therefore, effective treatment relies on appropriate diagnosis, whereas delay in diagnosis and treatment initiation can lead to greater morbidities and patient distress.

The large proportion of patients experiencing delay in time to first diagnostic procedure signals a quality concern and reflects an area for improvement. Several efforts may be beneficial. First, special attention is needed targeting patients particularly at risk for experiencing delay in PMB evaluation (such as patients at older age, with non-gynecologic comorbidities, or lacking preventive care as identified in our study). For instance, additional training for clinicians to be more vigilant about diagnostic evaluation of PMB in patients with competing health care needs of comorbidities may be helpful. Raising patients’ awareness about potential consequences of unattended PMB may improve adherence if follow-up visits are needed for evaluation. Second, clinicians should be cognizant of transportation and other potential care barriers and consider offering same day diagnostic procedures when feasible and acceptable to patient to promote timely evaluation. Third, there is growing evidence supporting potential usefulness of novel biomarkers and risk prediction algorithms for uterine cancer diagnosis [20–23]. In the long run, refinement and incorporation of these non-invasive evaluation tools may facilitate early diagnosis.

Insurance-based disparity in PMB evaluation observed in our study also has important public health and policy implications. Our finding is consistent with prior research in the United States showing that Medicaid patients were more likely than commercially insured patients to experience diagnostic and treatment delay for both cancer and non-cancer conditions [11, 24–27]. Since Medicaid patients have low economic resources, this raises concerns for equity in care quality. Moreover, in the United States, after the Patient Protection and Affordable Care Act was enacted in March 2010 (a health care reform law to expand insurance coverage and lower the costs of insurance coverage) [28], many Americans were able to gain insurance coverage via Medicaid expansion such that 17.8% of Americans were covered by Medicaid by 2020 [29]. Although expanding Medicaid insurance to more people helps improve access to and outcomes of care [30–32], acquiring Medicaid insurance alone may not be sufficient if there is inequity in care quality between Medicaid and commercial insurance. Further research is needed to identify effective quality improvement initiatives in Medicaid program, as well as strategies to address barriers to care in Medicaid (e.g., low reimbursement affecting providers’ willingness to accept Medicaid patients, more complex social and healthcare needs of Medicaid patients) [33–35].

We also identified other non-clinical factors that may increase a patient’s risk of having delayed evaluation, such as Black race and geographic location in the south or the west, pointing to unwarranted variation in quality of care which is another area needing additional research. Racial disparity in timeliness of care is well-documented in the literature, with Black patients generally more likely than White patients to experience delay in care for various conditions [36–38]. Two recent studies also reported that compared to White patients, Black patients with uterine cancer had a lower likelihood of receiving guideline-recommended diagnostic procedures [4, 5]. This can have important implications for patient outcome as certain aggressive histologic types of uterine cancer have a higher incidence among older and Black women (e.g., uterine serous carcinoma) [39, 40]. Our study expands this literature by demonstrating racial disparity in the diagnostic evaluation of PMB in the large number of women without cancer seeking care each year. Geographic variation in delayed evaluation observed in our study is also consistent with prior research reporting regional variation in utilization and outcomes of gynecologic care [41–43]. Nonetheless, the underlying reasons for these unwarranted variation in timeliness of diagnostic evaluation for PMB remain unclear and require further investigation. Such information is crucial for the design of remedy strategies.

Our inclusion of a large and diverse sample of patients with PMB in the United States (broad age range, both Medicaid and commercial insurance) helped enhance the generalizability of findings and our use of up-to-date nationwide data also helped depict contemporaneous practice patterns. Nonetheless, several study limitations should be acknowledged. First, we relied on insurance claims data to define PMB and other clinical measures, which may lack accuracy and granularity. For instance, we may miss the first reporting of PMB because patient-reported vaginal bleeding tends to be under-captured by administrative codes [44] and patients might switch insurance coverage after their initial PMB. However, that would suggest an even longer duration between initial PMB reporting and diagnostic evaluation, reinforcing concerns for delayed evaluation. We also lacked clinical detail on some risk factors for uterine cancer (such as history of tamoxifen use and hormone replacement therapy) that can confound clinical considerations for evaluation of PMB. In addition, due to potential under-coding of procedures/diagnoses in claims data and lack of insurance claims history beyond our study’s observation window, we may not identify all patients who had a prior hysterectomy. If some of those patients were not excluded from our sample, guideline-recommended diagnostic procedures examined in our study (which were geared towards evaluation of intrauterine pathology) may not be applicable to them. Second, we lacked information on patient sociobehavioral (e.g., transportation barriers) and provider-related (e.g., specialty) factors that may also influence timeliness of evaluation. Third, our sample did not include all patients with PMB. For instance, commercial insurance in the MarketScan Research Databases was limited to employer-sponsored health plans. Although employer-sponsored health insurance accounts for the largest share (54.4%) of Americans [14, 29], we lacked data on patients with self-purchased insurance or elderly patients without employer-sponsored retiree health benefit.

## Conclusions

Using large contemporaneous healthcare databases, we demonstrated a need to improve the timeliness of diagnostic evaluation for patients reporting PMB. Findings on the non-clinical factors that influence a patient’s risk of delayed evaluation also call for attention to unwarranted variation in care quality. Given the large number of women presenting with PMB each year [8], efforts to identify reasons for the inadequate evaluation of PMB and ways to improve care in this area are needed.

## Supporting information

S1 Table International Classification of Diseases (ICD) diagnosis/procedure codes, Current Procedural Terminology (CPT) codes, and Healthcare Common Procedure Coding System (HCPCS) codes used in defining relevant conditions and procedures. * indicates wildcard characters.
(DOCX)

S1 Fig Sample selection flow diagram. PMB = postmenopausal bleeding.
(DOCX)

S1 Checklist STROBE statement—Checklist of items that should be included in reports of observational studies. (DOCX)
