# Supplementary material for: Timeliness of diagnostic evaluation for postmenopausal bleeding: A retrospective cohort study using claims data
Source: PLoS One. 2023 Sep 8;18(9):e0289692. doi: 10.1371/journal.pone.0289692 (PMC10490884; doi:10.1371/journal.pone.0289692)
Supplement: S1 Table — * indicates wildcard characters. (DOCX) [file pone.0289692.s001.docx]

**S1 Table. International Classification of Diseases (ICD) diagnosis/procedure codes, Current Procedural Terminology (CPT) codes, and Healthcare Common Procedure Coding System (HCPCS) codes used in defining relevant conditions and procedures**

| **Diagnosis/Procedure** | **Codes** |
| --- | --- |
| ***Diagnosis*** |  |
| Uterine cancer | ICD-9: 179, 182.0-182.8  ICD-10: C54.0-C54.9, C55 |
| Other gynecologic cancer | ICD-9: 180.*, 181.*, 183.*, 184.*, 158.8, 158.9  ICD-10: C51.*-C53.*, C56.*-C58.*, C48.1, C48.2, C48.8 |
| Acquired absence of uterus | ICD-9: V88.01, V88.02  ICD-10: Z90.710, Z90.711 |
| Postmenopausal bleeding | ICD-9: 627.1  ICD-10: N95.0 |
| Leiomyoma | ICD-9: 218.*  ICD-10: D25.* |
| Benign neoplasms or cysts | ICD-9: 215.6, 219.*, 220, 221.*, 620.0, 620.1, 620.2, 621.0, 752.11  ICD-10: D21.5, D26.*, D27.*, D28.*, Q50.4, Q50.5, N83.0*, N83.1*,N83.2*, N84.* |
| Endometrial hyperplasia | ICD-9: 621.30, 621.31, 621.32, 621.33, 621.34, 621.35  ICD-10: N85.00, N85.01, N85.02 |
| Cervical abnormality | ICD-9: 233.1, 233.30, 233.31, 233.39, 622.10, 622.11, 622.12, 623.0, 795.00, 795.01, 795.02, 795.03, 795.04, 795.06, 795.09, 795.10, 795.11, 795.12, 795.13, 795.14, 795.16, 795.19  ICD-10: D06.0, D06.1, D06.7, D06.9, D07.2, D07.30, D07.39, N87.0, N87.1, N87.9, N89.3, R87.610, R87.611, R87.612, R87.613, R87.614, R87.618, R87.619, R87.620, R87.621, R87.622, R87.623, R87.624, R87.628, R87.629, R87.69 |
| ***Procedure*** |  |
| Hysterectomy | ICD-9: 68.3, 68.31, 68.39, 68.4, 68.41, 68.49, 68.5, 68.51, 68.59, 68.6, 68.61, 68.69, 68.7, 68.71, 68.79, 68.9  ICD-10: 0UT90*, 0UT94*, 0UT98*, 0UT9F *, 0UT97*  CPT: 58150, 58152, 58180, 58200, 58210, 58260, 58262, 58263, 58267, 58270, 58275, 58280, 58285, 58290, 58291, 58292, 58293, 58294, 58541, 58542, 58543, 58544, 58548, 58550, 58552, 58553, 58554, 58570, 58571, 58572, 58573, 58950, 58951, 58953, 58954, 58956 |
| Diagnostic procedure |  |
| Endometrial biopsy | ICD-9: 68.13  CPT: 58100, 58110 |
| Dilation and curettage | ICD-9: 69.09  CPT: 58120 |
| Histologic evaluation with  unspecified type (endometrial  biopsy or dilatation and curettage) | ICD-10: 0UDB7ZX, 0UDB7ZZ |
| Hysteroscopy | ICD-9: 68.12, 68.16  ICD-10: 0UDB8ZX, 0UDB8ZZ, 0UJD8ZZ  CPT: 58340, 58555, 58558, 58559, 58560, 58561, 58562, 58563, 58565 |
| Transvaginal/pelvic ultrasound | ICD-9: 88.79  ICD-10: BU46YZZ, BU46ZZZ, BU4CYZZ, BU4CZZZ  CPT: 76830, 76831, 76856, 76857 |
| Pelvic magnetic resonance  imaging | ICD-9: 88.95  ICD-10: BU36Y0Z, BU36YZZ, BU36ZZZ, BU3CY0Z, BU3CYZZ, BU3CZZZ  CPT: 72195, 72196, 72197 |
| Preventive care | CPT/HCPCS: 00812, 77063, 77067, 99381-99387, 99391-99397, 81528, 82270, G0101, G0104, G0105, G0106, G0120, G0121, G0123, G0124, G0141, G0143, G0144, G0145, G0147, G0148, G0202, G0327, G0328, G0344, G0402, G0438, G0439, G0468, G0476, P3000, P3001, Q0091 |

* indicates wildcard characters.
