# Supplementary material for: Timeliness of diagnostic evaluation for postmenopausal bleeding: A retrospective cohort study using claims data
Source: PLoS One. 2023 Sep 8;18(9):e0289692. doi: 10.1371/journal.pone.0289692 (PMC10490884; doi:10.1371/journal.pone.0289692)
Supplement: S1 Fig — PMB = postmenopausal bleeding. (DOCX) [file pone.0289692.s002.docx]

**S2 Fig. Sample selection flow diagram**

PMB = postmenopausal bleeding
