# Supplementary material for: Timeliness of diagnostic evaluation for postmenopausal bleeding: A retrospective cohort study using claims data
Source: PLoS One. 2023 Sep 8;18(9):e0289692. doi: 10.1371/journal.pone.0289692 (PMC10490884; doi:10.1371/journal.pone.0289692)
Supplement: S1 Checklist — (DOCX) [file pone.0289692.s003.docx]

STROBE Statement—checklist of items that should be included in reports of observational studies

|  | Item No. | Recommendation | Page  No. | Relevant text from manuscript |
| --- | --- | --- | --- | --- |
| **Title and abstract** | 1 | (*a*) Indicate the study’s design with a commonly used term in the title or the abstract | 1 | “A retrospective cohort study” |
|  |  | (*b*) Provide in the abstract an informative and balanced summary of what was done and what was found | 3-4 | “Methods: Using the 2008-2019 MarketScan Research Databases, we identified …” “Results: Overall, 54.3% of patients received a diagnostic procedure …” |
| Introduction | | | |  |
| Background/rationale | 2 | Explain the scientific background and rationale for the investigation being reported | 5-6 | “Postmenopausal bleeding (PMB) is a common condition, accounting for …” |
| Objectives | 3 | State specific objectives, including any prespecified hypotheses | 6 | “To address these knowledge gaps, we analyzed large healthcare databases in the United States to examine the timeliness of diagnostic evaluation in a large sample of patients who presented with PMB …” |
| Methods | | | |  |
| Study design | 4 | Present key elements of study design early in the paper | 7-10 | “We identified female patients who had at least one insurance claim with a diagnosis code of PMB and referred to their first PMB claim as the index PMB. Patients were included in this study if they met the following criteria …” |
| Setting | 5 | Describe the setting, locations, and relevant dates, including periods of recruitment, exposure, follow-up, and data collection | 6-10 | “We used the 2008-2019 IBM® MarketScan® Research Databases, which include a family of datasets integrating longitudinal claims data for a large sample of individuals with commercial insurance and Medicaid insurance in the United States …” |
| Participants | 6 | (*a*) *Cohort study*—Give the eligibility criteria, and the sources and methods of selection of participants. Describe methods of follow-up  *Case-control study*—Give the eligibility criteria, and the sources and methods of case ascertainment and control selection. Give the rationale for the choice of cases and controls  *Cross-sectional study*—Give the eligibility criteria, and the sources and methods of selection of participants | 7 | “We identified female patients who had at least one insurance claim with a diagnosis code of PMB and referred to their first PMB claim as the index PMB. Patients were included in this study if they met the following criteria …” |
|  |  | (*b*) *Cohort study*—For matched studies, give matching criteria and number of exposed and unexposed  *Case-control study*—For matched studies, give matching criteria and the number of controls per case | NA | NA (not a matched study) |
| Variables | 7 | Clearly define all outcomes, exposures, predictors, potential confounders, and effect modifiers. Give diagnostic criteria, if applicable | 7-9 | “Our outcome measure was the time from the date of a patient’s index PMB to the date when they received their first diagnostic procedure. This included …” |
| Data sources/ measurement | 8* | For each variable of interest, give sources of data and details of methods of assessment (measurement). Describe comparability of assessment methods if there is more than one group | 7-9 | “We measured each patient’s receipt of these diagnostic procedures from the date of their index PMB until 12 months afterwards using ICD and CPT procedure codes …” |
| Bias | 9 | Describe any efforts to address potential sources of bias | 10 | “To draw on additional measures of patient characteristics that were only available for commercially insured patients … or Medicaid patients … respectively, we also estimated separate Cox proportional hazards regressions stratified by insurance type …” |
| Study size | 10 | Explain how the study size was arrived at | S2 Fig | “Sample selection flow diagram” |

Continued on next page

| Quantitative variables | 11 | Explain how quantitative variables were handled in the analyses. If applicable, describe which groupings were chosen and why | 11 | “Age, in years  50-59  60-69  70-79  ≥80” |
| --- | --- | --- | --- | --- |
| Statistical methods | 12 | (*a*) Describe all statistical methods, including those used to control for confounding | 9-10 | “We estimated a multivariable Cox proportional hazards regression model to examine the association …” |
|  |  | (*b*) Describe any methods used to examine subgroups and interactions | 10 | “we also estimated separate Cox proportional hazards regressions stratified by insurance type and included these additional characteristics as covariates …” |
|  |  | (*c*) Explain how missing data were addressed | 9 | “Patients with missing data on a given characteristic were categorized as “unknown” and were retained in analysis.” |
|  |  | (*d*) *Cohort study*—If applicable, explain how loss to follow-up was addressed  *Case-control study*—If applicable, explain how matching of cases and controls was addressed  *Cross-sectional study*—If applicable, describe analytical methods taking account of sampling strategy | 8 | “Patients who did not receive any of these diagnostic procedures at the end of the observation period (i.e., 12 months after the index PMB) were considered censored.” |
|  |  | (*e*) Describe any sensitivity analyses | 10 | “we also estimated separate Cox proportional hazards regressions stratified by insurance type and included these additional characteristics as covariates …” |
| Results | | | | |
| Participants | 13* | (a) Report numbers of individuals at each stage of study—eg numbers potentially eligible, examined for eligibility, confirmed eligible, included in the study, completing follow-up, and analysed | S2 Fig | “Sample selection flow diagram” |
|  |  | (b) Give reasons for non-participation at each stage | S2 Fig | “Sample selection flow diagram” |
|  |  | (c) Consider use of a flow diagram | S2 Fig | “Sample selection flow diagram” |
| Descriptive data | 14* | (a) Give characteristics of study participants (eg demographic, clinical, social) and information on exposures and potential confounders | 11 | “Table 1. Sample characteristics” |
|  |  | (b) Indicate number of participants with missing data for each variable of interest | 11 | “Table 1. … Race and ethnicity … Unknown … Region … Unknown …” |
|  |  | (c) *Cohort study*—Summarise follow-up time (eg, average and total amount) | 8 | “Patients who did not receive any of these diagnostic procedures at the end of the observation period (i.e., 12 months after the index PMB) were considered censored.” |
| Outcome data | 15* | *Cohort study*—Report numbers of outcome events or summary measures over time | 12 | “Overall, 54.3% (271251/499176) of patients received a diagnostic procedure on the same day when they reported PMB and 86.6% (432073/499176) received a diagnostic procedure within 12 months after PMB reporting…” |
|  |  | *Case-control study—*Report numbers in each exposure category, or summary measures of exposure | NA | NA |
|  |  | *Cross-sectional study—*Report numbers of outcome events or summary measures | NA | NA |
| Main results | 16 | (*a*) Give unadjusted estimates and, if applicable, confounder-adjusted estimates and their precision (eg, 95% confidence interval). Make clear which confounders were adjusted for and why they were included | 13-18 | “In multivariable regression analysis for the overall sample, the association between type of insurance and time to first diagnostic evaluation remained statistically significant even after adjusting for patient age, …” |
|  |  | (*b*) Report category boundaries when continuous variables were categorized | 11 | “Age, in years  50-59  60-69  70-79  ≥80” |
|  |  | (*c*) If relevant, consider translating estimates of relative risk into absolute risk for a meaningful time period | NA | NA |

Continued on next page

| Other analyses | 17 | Report other analyses done—eg analyses of subgroups and interactions, and sensitivity analyses | 17 | “These associations remained similar when patients with commercial and Medicaid insurance were analyzed separately.” |
| --- | --- | --- | --- | --- |
| Discussion | | | | |
| Key results | 18 | Summarise key results with reference to study objectives | 18 | “We delineated patterns of diagnostic evaluation for non-cancer patients with PMB and found that a sizable proportion of patients did not receive prompt diagnostic evaluation after presenting with PMB ...” |
| Limitations | 19 | Discuss limitations of the study, taking into account sources of potential bias or imprecision. Discuss both direction and magnitude of any potential bias | 21 | “… several study limitations should be acknowledged. First, we relied on insurance claims data to define PMB and other clinical measures, which may lack accuracy and granularity …” |
| Interpretation | 20 | Give a cautious overall interpretation of results considering objectives, limitations, multiplicity of analyses, results from similar studies, and other relevant evidence | 18-22 | “In clinical practice for PMB, uterine cancer should be …” |
| Generalisability | 21 | Discuss the generalisability (external validity) of the study results | 21-22 | “our sample did not include all patients with PMB. … was limited to employer-sponsored health plans. …, we lacked data on patients with self-purchased insurance or elderly patients without employer-sponsored retiree health benefit.” |
| Other information | |  | | |
| Funding | 22 | Give the source of funding and the role of the funders for the present study and, if applicable, for the original study on which the present article is based | 2 | “Funding: This research was supported by the National Institute on Minority Health and Health Disparities of the National Institutes of Health under Award Number R01MD016386. ... The funder had no role in study design, data collection and analysis, decision to publish, or preparation of the manuscript.” |

*Give information separately for cases and controls in case-control studies and, if applicable, for exposed and unexposed groups in cohort and cross-sectional studies.

**Note:** An Explanation and Elaboration article discusses each checklist item and gives methodological background and published examples of transparent reporting. The STROBE checklist is best used in conjunction with this article (freely available on the Web sites of PLoS Medicine at http://www.plosmedicine.org/, Annals of Internal Medicine at http://www.annals.org/, and Epidemiology at http://www.epidem.com/). Information on the STROBE Initiative is available at www.strobe-statement.org.
